# OP54 Sequential Interventions And Cost-Minimization Analysis To Promote Disinvestment In Intravenous Immunoglobulin For Immune Deficiencies At A Pediatric Hospital

**DOI:** 10.1017/S0266462325101128

**Published:** 2025-12-29

**Authors:** Graciela Demirdjian, Marcela Rousseau

## Abstract

**Introduction:**

Immunoglobulin G (IgG) is a high-cost drug, leading our hospital pharmacy expense ranking since 2015, with a rising utilization trend from 16 percent of expenses in 2018 to 27 percent in 2023. The health technology assessment (HTA) unit at Hospital Garrahan proactively undertook sequential interventions to control variability, overutilization, costs, and resource use in the administration of IgG to pediatric patients with immune deficiencies. We report the results of a guideline implementation and a cost-minimization analysis.

**Methods:**

An evidence-based guideline on adequate IgG use was developed, indicating similar effectiveness for subcutaneous (SCIG) and intravenous IgG (IVIG) in replacement therapy for pediatric immune deficiencies. As the switch to SCIG was rejected due to higher cost, a sequential cost-minimization analysis was performed as SCIG hospital purchase prices decreased over time. Real-world data were collected from the hospital system on IgG units consumed, acquisition prices, and percentage of drug expenses from 2018 to 2023. Daycare bed occupation was calculated assuming monthly four-hour infusions for supplementary IVIG for all registered patients. Costs are expressed in Argentine pesos and equivalent American dollars.

**Results:**

In 2022, switching from IVIG to SCIG had an estimated annual budget impact of ARS51,189,073 (USD392,795); the proposal was rejected on cost grounds. The switch was recommended again in 2023 when prices of alternatives became equivalent. By 2024, the initial price situation reversed, and the change implied annual estimated savings of ARS4,413,487,361 (USD4.931.270). Sensitivity analysis considering price variations determined minimum annual savings of ARS1,408,175,937 (USD1,573,380). The switch was also estimated to free 432 daycare beds per year (1.8 beds/day) and 2,400 annual nurse work hours. The recommendation was finally accepted by managers and communicated to clinicians and specialists.

**Conclusions:**

Switching from IVIG to SCIG in pediatric immune deficiency patients can reduce hospital expenses, bed occupation, and nursing hours; additionally, it can prevent monthly hospital visits and subsequent school absenteeism. Hospital-based HTA can have a positive impact on cost reductions without sacrificing healthcare quality, especially by controlling adequate utilization of high-cost drugs and disinvesting in outdated technologies.

